# Predictive efficacy of immature granulocytes in acute complicated appendicitis

**DOI:** 10.1590/1806-9282.20241178

**Published:** 2024-12-02

**Authors:** Merve Yazla, Burcu Kadıoğlu, Hacer Demirdelen, Fatih Mehmet Aksoy, Erdem Özkan, Burak Katipoğlu

**Affiliations:** 1 Ankara Etlik City Hospital, Emergency Medicine – Ankara, Turkey.; 2 Ankara Etlik City Hospital, Clinic of Radiology – Ankara, Turkey.

**Keywords:** Acute appendicitis, Perforated appendicitis, Granulocytes

## Abstract

**OBJECTIVE:**

Acute appendicitis is the most common cause of acute abdomen. It is important to differentiate between complicated and uncomplicated appendicitis before surgery in the emergency department. Recently, immature granulocytes have become one of the biomarkers used as predictors of inflammation. The aim of this study was to determine whether immature granulocyte levels are a biomarker that can predict whether acute appendicitis is complicated or not in patients admitted to the emergency department.

**METHODS:**

Patients aged 18 years and older who presented to the emergency department between May 1, 2023, and April 30, 2024, and were diagnosed with appendicitis were included in the study. Patients with a histopathologic diagnosis of acute appendicitis were divided into two groups: acute simple appendicitis (n=149) and acute complicated appendicitis (n=103). Demographic characteristics, imaging results, and laboratory results were recorded.

**RESULTS:**

White blood cell, neutrophil count and percentage, lymphocyte count and percentage, immature granulocyte count and percentage, direct bilirubin, C-reactive protein, and procalcitonin values were found to be significantly higher in the complicated appendicitis group than in the uncomplicated group (p-values; 0.001, <0.001, <0.001, <0.001, 0.001, <0.001, <0.001, <0.001, <0.001, 0.016, <0.001, and 0.001, respectively). The immature granulocyte percentage was 92% specific for the diagnosis of complicated appendicitis at a cut-off value of 0.6.

**CONCLUSION:**

The immature granulocyte percentage may be useful as a predictive biomarker in the diagnosis of complicated acute appendicitis with a specificity of 92%. Additionally, the immature granulocyte percentage has a higher discrimination power than the immature granulocyte count, C-reactive protein, and procalcitonin.

## INTRODUCTION

Acute appendicitis (AA) is a disease characterized by bacterial colonization due to obstruction of the appendiceal lumen. It is the most common cause of acute abdominal pain requiring urgent surgical intervention in the emergency department (ED)^1^. Increasing the time between the onset of symptoms and surgical treatment is a serious risk factor for appendiceal perforation. Delays in diagnosis can lead to serious complications of appendicitis, such as perforation, abscess, or necrosis. This condition can cause serious morbidity and mortality^2^. Therefore, diagnostic markers for complicated acute appendicitis (CAA) have been investigated.

The use of computed tomography (CT) to determine whether a patient has complicated appendicitis is becoming increasingly common in clinical practice^3^. However, given the concerns regarding radiation exposure to the patient, as well as the workload and financial cost to radiologists, there remains a continuous search for a practical and cost-effective laboratory test that can predict this condition. Various biomarkers have been investigated to help predict whether AA is complicated^4^. However, their predictive value in distinguishing CAA is limited, and no consensus has been reached regarding their sensitivity and specificity.

Immature granulocytes (IG) are premature granulocytes released from the bone marrow in response to infection, inflammation, sepsis, and other stimuli. The automated counting of IG has been developed in hematology analyzers, which are fast, require no additional samples, and offer better reproducibility. Tests for IG can be performed simultaneously with the routine complete blood count (CBC) test^5^.

This study aims to evaluate the potential role of IG in the diagnosis of CAA in the ED by comparing them with C-reactive protein (CRP) and procalcitonin.

## METHODS

The study was designed as a prospective observational study. It commenced after obtaining approval from the Ethics Committee of Ankara Etlik City Hospital (AEŞH-EK1-2023-369). Written informed consent was obtained from all patients. The study was conducted in accordance with the Declaration of Helsinki.

Patients aged 18 years and older who presented to a tertiary ED, were diagnosed with appendicitis, underwent surgery, and were histopathologically confirmed to have appendicitis, were included in the study between May 1, 2023, and April 30, 2024.

The exclusion criteria were as follows: patients under 18 years of age, pregnant women, patients who did not have their initial blood tests performed at the current hospital or who were referred from an external center, patients with known immunological deficiencies or hematological disorders, patients on immunosuppressive or steroid therapy, patients who had received blood transfusions, patients with hematological malignancies, and patients with incomplete data.

Patient data were recorded in case forms with informed consent obtained from the patients. Patients with a histopathologically confirmed positive appendectomy who were operated on with a preliminary diagnosis of AA were included in the study. Based on histopathological examination reports, patients were divided into two groups: CAA and AA. Perforated appendicitis, localized or generalized peritonitis, acute phlegmonous appendicitis, acute gangrenous appendicitis, and abscess were classified as CAA, while patients diagnosed with AA were classified as uncomplicated appendicitis. IG and other markers obtained from tests performed during the first admission to the ED were compared between the CAA and AA groups.

### Sample size

In the sample size analysis conducted by considering the planned analyses separately, it was determined that a total of 210 participants would be sufficient for the t-test analysis to be performed with two groups, assuming an effect size of 0.5 (medium), an α value of 0.05, and a power of 0.95. It was also identified that the largest sample size requirement was for the t-test analysis. Accounting for a 20% dropout rate, it was planned to include 252 patients in the study. The sample size analysis was performed using the GPower 3.1 software.

### Statistical analysis

Descriptive statistics were presented as frequencies (percentages) for categorical variables and as means with standard deviations for numerical variables. The normality assumption for numerical variables was evaluated both analytically and graphically. Comparisons of patient characteristics and preoperative laboratory data between the CAA and AA groups were performed using the chi-square test for categorical variables and either the Independent Samples t-test or the Mann-Whitney U test for numerical variables. The diagnostic ability of laboratory parameters with statistically significant p-values from group comparison tests was assessed using the area under the receiver operating characteristic (ROC) curve. Cut-off values for potential biomarkers were determined using the Youden Index and diagnostic performance metrics, with sensitivity, specificity, and corresponding 95% confidence intervals, were calculated. Statistical analyses were conducted using the Statistical Package for Social Sciences (SPSS, Version 27, Inc., Chicago, IL) software, and a p<0.05 was considered statistically significant.

## RESULTS

A total of 555 patients were evaluated in the study, of which 252 patients with a histopathological diagnosis of appendicitis and no missing data were included. Of these patients, 64.3% were male. The mean age was 37.9±14.9 years (mean±SD). A diagnosis of AA was made in 149 patients, while 103 patients were diagnosed with CAA. Among those diagnosed with CAA, 29 had localized peritonitis, 38 had perforation, 16 had gangrenous appendicitis, 15 had phlegmonous appendicitis, 4 had plastron, and 1 had an abscess. The most common comorbid conditions were coronary artery disease (n=10), hypertension (n=7), and diabetes mellitus (n=7). The distribution of the patients’ sociodemographic data according to the groups is shown in Table 1.

**Table 1:** Sociodemographic characteristics of patients.

		AA 149 (59.1%)	CAA 103 (40.9%)	p-value
Gender, n (%)				
Male		90	72	0.122
Female		59	31	
Age (year)		33 (IQR 25–45)	39 (IQR 25–53)	0.053
Comorbidity		10 (14.9%)	14 (13.5%)	0.067
Symptom duration (h)		24 (IQR 10–48)	24 (IQR 12–48)	0.258
Imaging appendicitis thickness (mm)		10 (IQR 8–12)	12 (IQR 9.6–14)	0.002*
Imaging results	AA	142	12	<0.001*
	CAA	7	91	

AA: acute appendicitis; CAA: complicated acute appendicitis; IQR: interquartile range.

*Statistically significant values (p<0.05).

White blood cell (WBC), neutrophil count and percentage, lymphocyte count and percentage, IG count and percentage, direct bilirubin, CRP, and procalcitonin levels were significantly higher in the CAA group compared to the AA group. The distribution of patients’ laboratory data is shown in Table 2.

**Table 2:** Analysis of laboratory parameters.

Parameter	AA 149 (59.1%)	CAA 103 (40.9%)	p-value	Cohen’s d effect size**
WBC (μL/mL)	13.291±4.106	15.114±3.864	0.001	0.457
Hemoglobin (g/L)	13.9±2.3	14.5±1.8	0.201	
Neutrophil (%)	75±9	81±5	<0.001	0.824
Neutrophil	10.262±4.058	12.391±3.591	<0.001	0.555
Lymphocyte (%)	16±7	11±4	<0.001	0.877
Lymphocyte	2137±1167	1670±628	0.001	0.498
Platelet (×10^9^/L)	270.163±64.540	275.329±89.883	0.996	
IG %	0.371±0.178	0.448±0.189	<0.001	0.419
IG	0.05±0.03	0.07±0.03	<0.001	0.666
Urea	26±8	27±12	0.490	
Creatinine	0.8±0.1	0.9±0.5	0.075	
AST	23±20	25±9	0.788	
ALT	24±21	22±13	0.897	
Total bilirubin (mg/dL)	0.68±0.36	0.85±0.61	0.051	
Direct bilirubin (mg/dL)	0.24±0.12	0.29±0.16	0.016	0.353
CRP	38±45	68±79	<0.001	0.466
Procalcitonin	0.28±1.9	0.82±3.0	0.001	0.215
NLR	6.28±5.05	8.35±4.53	<0.001	0.431

AA: acute appendicitis; CAA: complicated acute appendicitis; AST: aspartate aminotransferase; ALT: alanine aminotransferase; CRP: C-reactive protein; IG:immature granulocytes; NLR: neutrophil to lymphocyte ratio; PLT: platelets; WBC: white blood cell.

**Cohen classified effect sizes as small (d = 0.2), medium (d = 0.5), and large (d≥0.8).

The diagnostic ability of laboratory parameters, performance measurements, and cut-off values are displayed in Table 3.

**Table 3:** Results of receiver operating characteristic analysis for laboratory parameters.

	AUC (95%CI)	p-value	Cut-off value	Sensitivity (95%CI)	Specificity (95%CI)	PPV	NPV
WBC (μL/mL)	0.621 (0.578–0.717)	0.001	12,980	70%	53%	51%	72%
Neutrophil (%)	0.672 (0.605–0.740)	<0.001	76	75%	56%	54%	77%
Neutrophil	0.646 (0.577–0.716)	<0.001	10,870	62%	64%	54%	71%
Lymphocyte (%)	0.323 (0.256–0.391)	<0.001	–	–	–	–	–
Lymphocyte	0.380 (0.310–0.451)	0.001	–	–	–	–	–
IG (%)	0.648 (0.578–717)	<0.001	0.6	23.5%	92.6%	68%	63%
IG	0.677 (0.610–0.745)	<0.001	0.05	78%	49%	51%	77%
D. BIL	0.589 (0.516–0.661)	0.018	0.19	76%	37%	45%	69%
CRP	0.619 (0.546–0.691)	0.002	53	48%	78%	60%	68%
Procalcitonin	0.607 (0.535–0.680)	0.004	0.1	48%	72%	54%	67%
NLR	0.677 (0.609–0.745)	<0.001	4.81	81%	52%	54%	80%

AUC: area under curve; CI: confidence interval; NPV: negative predictive value; PPV: positive predictive values; p<0.05 is statistically significant. AA: acute appendicitis; CAA: complicated acute appendicitis; CRP: C-reactive protein; D.BIL: direct bilirubin; IG: immature granulocyte; NLR: neutrophil-to-lymphocyte ratio; WBC: white blood cells.

## DISCUSSION

AA is the most common surgical emergency among patients presenting with abdominal pain to the ED. Distinguishing whether appendicitis is complicated in the ED is important for planning treatment, informing the patient, and predicting what the surgeon will encounter during surgery. IG, a parameter that can be obtained from a CBC, is simple, inexpensive, and easily accessible in all patients. Our study has identified that with a cut-off value of 0.6, IG% can predict CAA with 92% specificity. Therefore, we believe that the IG% can help predict complicated appendicitis in patients diagnosed with AA.

The advent of advanced imaging techniques such as CT has increased the diagnostic sensitivity for CAA; however, the use of CT is costly and cumbersome. Furthermore, it should not be used in children, pregnant women, or patients with a glomerular filtration rate ≤30 mL/min^6^. Biochemical markers are a promising tool for identifying CAA. Many studies have noted that while WBC, neutrophil, lymphocyte, and neutrophil-to-lymphocyte ratio (NLR) values are sensitive for AA, they lack specificity and demonstrate low sensitivity in distinguishing between complicated and uncomplicated cases^7^. In our study, consistent with the literature, diagnostic variables for these biomarkers were assessed, and a specificity rate of 65% was not surpassed.

During the inflammation process, the effect of pro-inflammatory cytokines initiates an increase in the synthesis of CRP from the liver. CRP levels begin to rise within 4–6 h of inflammation and reach peak levels within 24–48 h^8^. While the rapid increase in CRP levels is advantageous, its wide range of sensitivity (40–94%) and specificity (38–87%) presents a disadvantage^9^. Numerous studies have demonstrated that elevated CRP levels are a valuable and usable marker for distinguishing between AA and CAA, but it is not specific^4,10^. In our study, CRP levels above 53 mg/dL were statistically significantly associated with CAA with a specificity of 78%. However, it did not provide high enough diagnostic validity to be used alone in practice. Additionally, analyzing CRP as a separate parameter from CBC incurs additional costs.

Compared to CRP, the value of procalcitonin in predicting CAA has been less evaluated, and the results are controversial. While there are studies showing very high accuracy for procalcitonin (with an ROC AUC of 0.987), there are also recent studies demonstrating low performance (ROC AUC: 0.59)^11,12^. In our study, procalcitonin showed weak accuracy (with an ROC AUC of 0.607) and remained at a low sensitivity level. Not only will its diagnostic use alone be insufficient, but similar to CRP, analyzing procalcitonin as a separate parameter from the CBC will result in extra costs.

In advanced stages of appendicitis, particularly after perforation, the development of hyperbilirubinemia has been shown in cases of necrosis and gangrene. Bacterial endotoxins inhibit the release of bilirubin into the bile ducts, subsequently causing an increase in serum bilirubin^13^. A meta-analysis examining the impact of high serum bilirubin levels on the likelihood of perforated appendicitis reported a sensitivity of 49% and a specificity of 82%. It was concluded that, despite some predictive potential of high total bilirubin levels, hyperbilirubinemia alone cannot be used to distinguish perforated appendicitis^14^. In our study, it was observed that bilirubin levels increased in CAA, but it was only statistically significant. This confirms, in line with the literature, that elevated bilirubin alone is not a reliable biomarker for the diagnosis of CAA in the ED.

In recent years, new-generation hemogram devices have been capable of calculating the IG and IG%, thereby serving as an easily accessible and cost-effective marker^15,16^. Studies utilizing IG as a predictor of CAA have observed a wide range of sensitivity and specificity values. These inconsistencies may be due to many studies being designed retrospectively, the presence of comorbidities, or variations in symptom duration. Differences in sample size and cut-off values might also contribute to the variability in reported outcomes. Thus, the diagnostic efficacy of IG as a predictor of CAA in emergency settings has not yet been fully established. In a study by Turkes et al., the count of IG was shown to be a strong negative predictive test for AA disease and more predictive than another CBC test for CAA^17^. The study was designed retrospectively and analyzed a total of 99 patients. Although IG% provided a discriminatory advantage in our study, conversely, our study cautions against over-reliance on these parameters to confirm the diagnosis of CAA due to their low specificity and positive predictive value. A recent review encompassing 65 laboratory tests supports this view^18^, concluding that no single biomarker can be used by clinicians to differentiate between patients with CAA and AA.

Our study is examined from a methodological and statistical perspective; It was designed as a prospective observational study. It is a study with a sufficient sample size with 95% power. Since inflammatory biomarkers observed in appendicitis patients were consistently higher than healthy controls in various studies, a healthy control group was not included in our study to avoid unnecessary workload and cost increase. Statistical significance examines the probability of findings being due to chance. Effect size helps readers understand the magnitude of differences found, not just whether a treatment affects people but how much it impacts them. Therefore, effect sizes are also provided in our study. Procalcitonin and direct bilirubin showed a small magnitude of effect; WBC, neutrophil, lymphocyte, IG%, CRP, and NLR demonstrated a medium magnitude of effect. Neutrophil%, lymphocyte%, and IG have a high magnitude of effect. We recommend that researchers take these into consideration.

In this study, patients who were operated on for appendicitis but received a non-appendicitis diagnosis upon pathological examination were excluded. This exclusion ensured that only patients with a histopathologically confirmed diagnosis were included in the study, thereby preventing a significant limitation that could have arisen in our research. Nonetheless, our study has some limitations. The first is its nature as a single-center observational study. Second, no clinical scoring system was included to compare the biomarkers tested. Additionally, clinical symptoms and physical examinations were not analyzed in this study.

## CONCLUSION

IG% may be useful as a predictive biomarker for CAA with a specificity of 92%. The discriminatory power of IG% is higher than that of IG, CRP, and procalcitonin. Despite significant differences being detected between the two groups for IG, CRP, and procalcitonin, the diagnostic ability to identify CAA was weak for all three biomarkers, which may limit their clinical utility.
